# Infectious Complications after Umbilical Cord-Blood Transplantation from Unrelated Donors

**DOI:** 10.4084/MJHID.2016.051

**Published:** 2016-10-18

**Authors:** Juan Montoro, José Luis Piñana, Federico Moscardó, Jaime Sanz

**Affiliations:** Hematology Department, University Hospital La Fe and Department of Medicine, University of Valencia, Valencia, Spain

## Abstract

Umbilical cord-blood (UCB) is a well-recognized alternative source of stem cells for unrelated donor hematopoietic stem cell transplantation (HSCT). As compared with other stem cell sources from adult donors, it has the advantages of immediate availability of cells, absence of risk to the donor and reduced risk of graft-versus-host disease despite donor-recipient HLA disparity. However, the use of UCB is limited by the delayed post-transplant hematologic recovery due, at least in part, to the reduced number of hematopoietic cells in the graft and the delayed or incomplete immune reconstitution. As a result, severe infectious complications continue to be a leading cause of morbidity and mortality following UCB transplantation (UCBT). We will address the complex differences in the immune properties of UCB and review the incidence, characteristics, risk factors, and severity of bacterial, fungal and viral infectious complications in patients undergoing UCBT.

## Introduction

Over the past decades, remarkable progress has been made in several areas of allogeneic hematopoietic stem cell transplantation (allo-HSCT). In particular, the use of alternative sources of progenitors stem cells, such as unrelated donors (URD), HLA-haploidentical family donors and umbilical cord-blood (UCB) grafts, has extended the transplant procedure to almost every patient in need of an allo-HSCT. Umbilical cord blood transplantation (UCBT) has rapidly become a valuable alternative for adult patients who lack a well HLA-matched donor providing similar outcomes compared to those allografted from URD in patients with hematological malignancies.^1^,^2^,^3^ The lower requirements for HLA-matching between UCB grafts and recipients, the significantly faster availability of banked cryopreserved UCB units compared to other sources of allo-HSCT, the lower incidence of graft-versus-host disease (GVHD) while preserving the graft-versus-leukemia effect, and the lower risks of viral or other infectious disease transmission make UCB an attractive source of stem cells progenitors, particularly pertinent for those patients requiring urgent transplantation.^4^ In addition, advances in UCB unit selection citeria, an essential aspect of UCBT, have led to a continuous improvement in the overall survival over the last years.^5^

Unfortunately, UCBT has several limitations, in particular, those associated with the prolonged period of neutropenia and the delayed immune reconstitution that has been related with a high transplantation-related mortality rate, up to 50% in several historical series.^1^,^2^ As a result, infections remain the leading cause of morbidity and mortality during the first six months after transplantation.^6^,^7^,^8^,^9^,^10^

The infection patterns after an allo-HSCT are classically divided into 3 periods according to the immunodeficiency status of the recipients. These periods show marked differences among the characteristics and incidence of infections.^11^ Briefly, the first phase comprised from day 0 to day +30, the intermediate from day +30 to +100 and the later beyond day +100. So far, it has been suggested that UCBT may follow these well-known differentiated patterns.^12^ However, intrinsic characteristics related to this procedure such as the naïve immune system status of the recipient or the low nucleated and CD34^+^ cell dose may vary the pattern of the infectious complications when compared to other stem cell sources. In view of these concerns, several strategies have been explored to enhance engraftment and shorten neutropenia, such as the infusion of double-cord blood units, CD34+ cells *ex-vivo* expansion or co-infusion of CD34+ cells from a third-party donor. Regrettably, none of these new approaches has shown clear benefits compared with using a single un-manipulated cord blood unit.^13^,^14^,^15^,^16^

To date, few studies have analyzed the infectious complications among adult UCBT recipients. Here we review the incidence, characteristics, risk factors, and severity of bacterial, viral and fungal infectious complications in the UCBT setting reported in the literature.

## Particularities of the immune function after UCBT

As compared to allogeneic bone marrow transplantation and peripheral blood stem cell transplantation, UCBT has unique and inherent immunological properties and peculiarities.

First, the transmission of cord-blood-naïve T lymphocytes shows gradual expansion in response to antigens, higher threshold for stimulation by cytoquines and low cytotoxic ability.^17^,^18^ Also, the potential benefit of passively transferring the humoral immunity from the donor to the recipient is lacking and may increase the risk of severe infections in the early period. Reconstitution of the T-cell compartment after UCBT is a slow process that can extend beyond the first year after transplant. T-cell recovery follows 2 different pathways with distinct kinetics: the thymus-independent pathway and the transferred donor T-cell or the recipient T cells that survive conditioning.^19^,^20^ In essence, delayed immune reconstitution after UCBT is characterized by prolonged T-cell lymphopenia, compensatory recovery, and expansion of B and natural killer cells, impaired functional T-cell responses and thymopoietic regenerative failure associated with late memory T-cell skewing.^21^ Also, UCB grafts contain relatively more CD4^+^CD25^+^ T regulatory cells (Treg) that may exert a more potent suppressor function than those from adult peripheral blood.^22^

Second, the use of anti-thymocyte globulin (ATG) as a component of some preparative regimens to prevent graft rejection influences the number and function of both, the central and the peripheral T cells infused within UCB grafts.^21^ In this sense, several studies have observed a higher risk of infection with the use of ATG for UCBT, especially for viral infections.^21^,^23^,^24^,^25^,^26^ In contrast, a recent comparative study in children with acute lymphoblastic leukemia showed no increased risk of infection or mortality related to the administration of ATG as a part of the conditioning regimen.^27^ A detailed prospective assessment of immune reconstitution was undertaken in adult patients who underwent double UCB-HSCT with ATG or peripheral blood HLA-matched URD transplantation after a reduced-intensity conditioning regimen.^28^ They showed that reconstitution of CD3^+^ T cells, including naive (CD45RO^−^) and memory (CD45RO^+^) CD4^+^ T cells, regulatory (CD4^+^CD25^+^) T cells, and CD8^+^ T cells was significantly delayed in the UCBT group. These findings suggested that increased risk of infections were specifically associated with a delayed reconstitution of all major T cell subsets and, interestingly, without an increased risk of relapse, suggesting that graft-versus-leukemia activity may be maintained by the early reconstitution of B cells and NK cells.

Finally, the low number of hematopoietic stem cells in UCB units has been associated with a higher incidence of infections because of a delayed neutrophil recovery.^2^,^7^,^29^,^30^ However, when selecting an adequate single unit (conventionally defined as at least 2.5×10^7^ nucleated cells/kg),^31^ episodes, severity and deaths related to infections, seemed similar than with other stem cell sources 1,15

In conclusion, more studies to improve the understanding of immune recovery after UCBT are needed.

## Bacterial Infections

Severe bacterial infections remain a leading cause of morbidity and non-relapse mortality, especially before day 100 in patients undergoing UCBT.^12^,^32^,^33^ However, few studies have specificaly adressed this issue in the UCBT setting.^30^,^33^,^34^,^35^ Some of these studies have included a relatively small number of patients with few documented infections or have reported bacterial infections together with other severe infections.^6^,^12^,^13^,^14^,^32^,^36^,^37^ It is, therefore, difficult to draw definite conclusions from these studies and efforts to better define the epidemiology, clinical characteristics, outcome and prognostic factors of bacterial infections after UCBT are warranted.

Reported incidence of bacterial infections ranged from 12% to 64% depending on the follow-up and the intensity of the conditioning regimen. Studies that were focused in the early/intermediate period (until day +100) reported an incidence ranging from 30 to 40%.^32^,^34^ However, with longer follow-up, the incidence increased up to 50 to 70% at 4 years.^30^,^32^ Interestingly, a bimodal distribution of baterial infections following UCBT has been proposed, with 25–40% episodes occurring within the first month and 25–30% after 100–180 days postransplant.^32^,^36^ These data suggest an increased predisposition to late infections after UCBTrelated to the delayed immune reconstitution and/or the profound immunosuppression status derived from the GVHD and/or its treatment.^38^

Most studies have demonstrated a predominance of Gram-positive bacteria (GPB) bloodstream infections (BSI) occurring before day +100^6^,^33^,^34^,^35^ and in others within the first year post-transplant.^14^ Among the GPB, *coagulase-negative* s*taphylococcus* (CoNS) were more common*,* especially before engraftment due to catheter-related infections (66% of CoNS), followed by *Enterococcus species.* Indeed, we also observed that the type of GPB infections differed significantly with longer follow-up observation time. However, we recently showed that overall Gram-negative rods (GNR) bacteremia in UCBT recipients was more common than GPB, with a ratio of 1.6.^30^,^32^ Among GNR, *Escherichia coli* and *Pseudomonas spp*. were the most frequently isolated bacteria (32% and 29% of GNR, respectively).^30^ Limited retrospective data suggest higher rates of BSI after UCBT compared to T-cell depleted haploidentical^36^ or URD peripheral blood/bone marrow transplants.^32^,^37^ These differences were restricted to the first 100 days after transplantation, however, beyond 100 days the incidence of BSI was comparable without differences in term of bacteremia-related mortality between stem cell sources.^32^,^37^ Several studies have identified risk factors associated with increased risk of infections after UCBT depicts the profound immunosuppression status. The length of neutropenia, the delayed lymphocyte recovery, the low cell dose content of the graft, such as total nucleated cells, CD34^+^ and CD8^+^ cells are frequently observed in this scenario.^30^,^32^, ^37^

BSI in the UCBT has a negative impact on mortality. The death rate of bacterial infections in the early postransplant period is around 25%, mostly due to GNR. Most frequent reported organisms causing fatal BSI were *Acinetobacter* spp., followed by *Stenotrophomonas maltophilia, Klebsiella-Enterobacter-Serratia, Pseudomonas aeruginosa and Escherichia coli*.^30^,^34^ Early BSI (before day +7) significantly delayed neutrophil recovery and was an independent risk factor associated with non-relapse mortality.^30^,^33^

Table 1 summarizes bacterial infections characteristics in the UCBT published series.

The delayed immune recovery, typically associated with the UCBT, is the primary limiting factor. Thus, there is an urgent need for developing strategies that overcome these conditions. To date, the only procedure that has demonstrated to shorten neutropenia is so called “haplo-cord” transplants in which CD34^+^ selected cells from the mobilized peripheral blood of an HLA-mismatched third-party donor are co-infused with the UCB unit. However, there were no differences in the rate of bacterial infection-related mortality post transplantation suggesting that such a benefit might not be clinically relevant.^13^

As a summary, bacterial infections are a major complication following UCBT particularly in the early/intermediate period. There are few but relevant differences in the incidence and characteristics of BSI in UCBT compared to other stem cell source. As in other procedures, BSI have an adverse impact on NRM in UCBT. Thus, further strategies focused on improving immune reconstitution are of utmost importance in this setting.

## Fungal Infections

Again, studies focusing on invasive fungal infections (IFIs), both invasive candidiasis and invasive mold infections after UCBT are scarce. Described incidence varies among different transplantation centers depending on many factors such as the geographical region, patients diagnosis, conditioning, period of neutropenia and type of primary prophylaxis.^7^,^12^,^13^,^14^,^21^,^30^,^32^,^36^,^37^,^39^–^43^

Reported incidences of fungal infections ranged from 10% to 38%, of which 33%-100% occurred before day +50. Most common sites of IFI were invasive pulmonary infections followed by brain abscess and disseminated fungal infection. Of the sixty-three documented fungal infections published in the literature, 35 episodes of fungemia were caused by *Candida*, 19 by *Aspergillus*, 2 by *Scedosporium*, 2 by Zygomycetes, 1 by *Cryptococcus, 1 by Saccharomyces,* 1 by *Fusarium,* 1 by *Trichosporon* and 1 by Rhizopus.^6^,^12^,^13^,^14^,^30^,^36^,^37^,^41^,^42^ A higher proportion of IFIs before day +100 after UCBT compared to bone marrow or peripheral blood stem cell transplantation has been suggested.^32^ However, in the long term, the 3-year cumulative incidence risk of developing an IFI in 192 patients was 12% and did not differ between stem cell sources. *Candida* was the most common fungal pathogen during the pre-engraftment period with an incidence of 2% and a median time to onset of 48 days (range, 4–122). Interestingly, all cases were due to non–*Candida albicans* species. No risk factors were found for *Candida* infection, except for a trend in cases of prolonged neutropenia beyond day +30. On the other hand, severe aGVHD, use of prednisone and delayed neutrophil recovery have been identified as risk factors for early invasive aspergillosis.^32^,^41^

More recently, advances in antifungal prophylaxis have diminished the incidence of IFIs in the early postransplant period and have resulted in a delayed onset, associated with GVHD and use of corticosteroids or other immunosuppressive therapies.^40^,^44^ The incidence of late invasive fungal episodes (after day + 100) ranges from 10% to 66% with a time to diagnosis ranging from 148 to 1350 days 37,40,44. Risk factors for late IFIs were acute and chronic GVHD, use of ATG for the treatment of severe GVHD and the use of nonmyeloablative conditioning regimen. 14,37,40,44

Finally, no differences have been found in outcome after IFI, with a similar mortality rate after UCBT or transplant with other stem cell sources ranging from 7% to 86%.^13^,^21^,^30^,^32^,^36^,^41^,^43^

Table 2 summarizes fungal infections characteristics in the UCBT published series.

## Viral Infections

The UCB graft is pathogen-naïve, and infused T cells are considered antigen-inexperienced. Therefore UCBT recipients are at increased risk of severe viral infectios when compared with other graft sources, specially due to viral pathogens that require a strong T-cell immunity control such as for the herpes viruses family.^18^,^45^,^46^,^47^,^48^,^49^ In the current review, we summarize the most relevant viral infections in the UCBT setting: cytomegalovirus (CMV), Epstein-Barr virus (EBV) and human herpes virus-6 (HHV-6).

### CMV

atients receiving UCBT are at high risk of CMV infection and disease because of the poor T-cell mediated cellular immune reconstitution.^50^ Nevertheless, the incidence, outcome, and risk factors for CMV infection and illness after UCBT have been barely addressed and vary considerably across transplantation centers.^48^,^51^–^60^ Higher rates of CMV infection and disease have been observed in seropositive recipient experiencing acute or chronic extensive GVHD.^52^

Only two retrospective studies have compared CMV infection and illness in UCBT or other stem cell sources.^52^,^58^ Walker and colleagues evaluated episodes of CMV infection in 753 consecutive allo-HSCT patients. The 6-month cumulative incidence of CMV infection and disease in UCBT recipients was 21% and 6%, respectively. These results were very similar to those observed in recipients of peripheral blood or marrow grafts. Interestingly, CMV infection did not have an adverse impact on survival.^52^ The study carried out by Mikulska et al., compared CMV infection in 80 UCBT recipients and 85 unrelated matched or mismatched donors. A higher incidence of late CMV infection and a longer duration of infections was seen in UCBT patients when compared with adult donor transplants in which donor and recipient were seropositive. No difference in mortality was observed.^52^,^58^

The relative efficacy of universal prophylaxis compared to a preemptive approach’s hard to establish. CMV-seropositive patients not receiving prophylaxis had a high incidence of CMV infection, ranging from 70% to 100% at day +100, along with an earlier presentation.^52^–^55^,^60^ The incidence of CMV infection ranged from 41% to 79% in studies using prophylaxis with high-dose acyclovir, ganciclovir or valganciclovir.^48^,^52^,^56^,^61^–^63^ The incidence of CMV disease after UCBT ranged from 1% to 18% according to differences in conditioning regimens, methods of CMV prevention, patient characteristics, and other variables.^48^,^52^,^53^,^54^,^57^,^60^,^63^ An incidence of CMV disease of 16% at day +100 was reported using preemptive therapy with ganciclovir or foscarnet in a series of 140 UCBT recipients.^55^ Another study from our group using prophylaxis, the risk of CMV disease in CMV-seropositive recipients was 3% at day +100 but increased to 12% at 1 year after transplantation.^51^ This finding is consistent with previous studies and supports the hypothesis that CMV prophylaxis can delay recovery of CMV-specific T cell immunity.^64^ We also showed similar overall survival, non-relapse mortality, and infection-related mortality in CMV-seropositive and -seronegative patients, suggesting that prophylaxis with either intravenous ganciclovir or oral valganciclovir may overcome the survival disadvantage of CMV-seropositive patients in the setting of UCBT.^63^

### EBV

EBV reactivation and EBV associated post-transplantation lymphoproliferative disorder (EBV-PTLD) does not seem to be increased in myeloablative UCBT compared to other unrelated donor transplants. The reported incidence of EBV viremia or PTLD in this setting is around 3–5%.^23^,^49^,^65^ However, EBV related complications increase exponentially after UCBT using ATG in reduced intensity conditioning regimens with a reported incidence of EBV viremia and PTLD of 18% and 13%, respectively.^23^,^49^,^65^–^68^ In addition, despite the fact that studies are heterogeneous with respect to patients, disease, and transplant characteristics, a higher incidence of EBV-PTLD has been suggested in patients with Hodgkin’s disease.^65^,^68^ The clinical presentation of PTLD may also be somewhat different showing early onset (median time of 75 days), frequent disseminated disease and extranodal involvement commonly affecting liver and spleen.^49^,^65^,^66^ The clinical course was aggressive with high mortality despite the administration of rituximab or chemo-immunotherapy when feasible.^65^,^67^–^69^

### HHV-6

HHV-6 reactivation is frequent after UCBT and can be detected in over 80% of patients early after transplantation.^45^,^70^–^74^ The clinical significance of viral reactivation is unknown, although it has been associated with many complications including encephalitis, marrow suppression and delayed engraftment, skin rash, hepatitis, interstitial pneumonia and an increased risk of developing aGVHD. A recent meta-analysis showed an increased prevalence of HHV-6 reactivation and severity of HHV-6 associated disease in patients receiving UCBT in comparison to other stem cell sources, recommending a closely monitoring for HHV-6 reactivation in this setting.^75^ A more recent study investigated HHV-6 reactivation within 60 days of transplantation in stem cell transplants using single UCB, double UCB, or UCB plus haploidentical stem cells. Of 92 patients, 60 (65%) had HHV-6 reactivation. Reactivation was not significantly influenced by any patient characteristics, disease characteristics, or by stem cell source. Indeed, they did not observe any impact of HHV-6 reactivation on neutrophil or platelet count recovery or on relapse-free survival. However, HHV-6 reactivation was associated with subsequent development of acute GVHD (HR = 3.00; 95% CI, 1.4 to 6.4; p = 0.004).^76^

## Conclusions

Over the past three decades, remarkable progress has been made in the use of UCB as an alternative stem cell source for allogeneic transplantation for patients lacking a suitable HLA-matched donor. However, UCBT is still limited by the low cell dose of the graft and the slow or incomplete immune reconstitution, resulting in a high transplantation-related mortality (TRM) due to infections. It’s hard to compare the impact on infection risk of UCB with other transplant strategies since there are no randomized studies. The sequential post-transplant periods: early (<30 days), intermediate (days +30 to +100) and late (days > +100) are associated with characteristic patterns of infectious complications after UCBT. Efforts to improve graft selection, shorten neutropenia, enhance immune reconstitution and develop prevention and supportive care measures are wanted and should be the primary focus of clinical research in the field.

## Figures and Tables

**Table 1:** Bacterial infections characteristics in the UCBT published series.

Reference	No of patients	Study period	Incidence of bacterial infections in each period, %			Organisms, %		Bacterial related mortality, %
			Early	Intermediate	Late	Gram-negative	Gram-positive	
Cahu - 2009^12^	31	2003–2008	16	9	16	42	57	0
Parody - 2006^32^	48	1997–2005	31	7	32	84	16	29
Yazaki - 2009^33^	1872	1997–2005	9–19	11–21	nr	26	74	16–30
Sanz - 2015^30^	241	1997–2012	34	42	52–54	51	42	12–23
Narimatsu - 2005^34^	102	2002–2004	25	32	nr	41	59	25
Tomonari - 2007^35^	101	1998–2006	12	nr	nr	33	67	17
Saavedra - 2002^6^	27	1997–2001	nr	63	nr	39	61	11
Sauter - 2011^14^	72	2005–2009	62	52	50	31	59	0
Mulanovich - 2011^36^	37	2001–2008	43	31	nr	15	85	nr
Hamza - 2004^37^	28	1998–2003	3	nr	nr	15	85	nr

Abbreviations: nr indicates not reported

**Table 2:** fungal infections characteristics in the UCBT published series.

Reference	No of patients	Study period	Fungal infections, %	Fungal pathogen, %		
				Aspergillus spp.	Candida spp.	Other
Long - 2003^7^	57	1996–2002	45	nr	nr	nr
Cahu - 2009^12^	31	2003–2008	10	100	0	0
Martino - 2015^13^	77	2000–2010	26	80	20	0
Sauter - 2011^14^	72	2005–2009	14	0	0	100
Sanz - 2015^30^	241	1997–2012	7	nr	93	7
Parody - 2006^32^	48	1997–2005	12	58	42	0
Mulanovich - 2011^36^	37	2001–2008	30	36	46	18
Hamza - 2004^37^	28	1998–2003	32	33	11	56
Miyakoshi - 2007^41^	128	2002–2005	11	nr	nr	7
Ferrá - 2010^42^	62	2000–2007	17	nr	nr	nr
Montesinos - 2015^43^	36	2001–2003	51	88*	4*	9*
Saavedra - 2002^6^	27	1997–2001	11	1	3	0
Almyroudis - 2009^44^	15	1997–2007	26	3	1	0
